# Association between Serum 25-Hydroxyvitamin D Level and Rheumatoid Arthritis

**DOI:** 10.1155/2015/913804

**Published:** 2015-05-04

**Authors:** Xiaomin Cen, Yuan Liu, Geng Yin, Min Yang, Qibing Xie

**Affiliations:** ^1^Department of Rheumatology and Immunology, West China Hospital, Sichuan University, Chengdu, Sichuan 610041, China; ^2^Department of Rheumatology and Immunology, First Affiliated Hospital of Xiamen University, Xiamen, Fujian 361003, China

## Abstract

The objective of this study is to examine and evaluate whether serum 25(OH)D is associated with disease activity in patients with rheumatoid arthritis (RA). Our results suggested that serum 25(OH)D in RA groups has significant lower level (35.99 ± 12.59 nmol/L) than that in the normal groups (54.35 ± 8.20 nmol/L, *P* < 0.05). Based on the DAS28, patients with RA were divided into four subgroups, and no differences were found in the four groups (*P* > 0.05). The 25(OH)D levels in complete remission, low disease activity, middle disease activity, and high disease activity group were 32.86 ± 12.26, 33.97 ± 13.28, 38.41 ± 10.64, and 38.94 ± 13.35 nmol/L, respectively. Based on the serum 25(OH)D levels, patients with RA were divided into inadequate group and normal group, and there were no significant differences in baseline characteristics and disease activity in the two groups. Our results showed that serum 25(OH)D levels in the inadequate group are significantly lower than those in the normal group. However, no correlations were found between 25(OH)D levels and disease activity among 116 patients with RA. The present findings will help to understand the association between 25(OH)D and disease activity of RA.

## 1. Introduction

Vitamin D has important functions in physiological processes, and vitamin D deficiency may play a role in pathophysiological processes and has increasingly been recognized as an important human health problem. 25-hydroxyvitamin D [25(OH)D], the accepted measure of vitamin D status, has been linked to several pathological states, including cardiovascular diseases, inflammatory disease, and high all-cause mortality in the general population [1]. Recent studies showed that 25(OH)D not only regulates calcium and phosphorus metabolism, but also plays a role in regulating immune and anti-inflammatory activities by adjusting growth and differentiation of macrophages, dendritic cells, T lymphocytes, and B lymphocytes, inhibiting inflammatory factors such as TNF-*α*, and promoting generation of anti-inflammatory factors such as IL-4 and IL-10 [2, 3].

Rheumatoid arthritis (RA) is a chronic inflammatory autoimmune disease characterized by synovitis. The aetiology and pathogenesis of RA remain obscure and many factors may be associated with its pathogenesis. 25(OH)D has immunoregulatory activities, so it is an important factor that may increase the prevalence of many autoimmune diseases such as RA and systemic lupus erythematosus [4, 5]. Increasing findings also supported that low or deficient levels of 25(OH)D may be associated with an increased risk for the development of RA [5–7]. Recently, the role of vitamin D deficiency in the pathogenesis of RA, as well as the relationship between vitamin D deficiency and the activity of RA, is discussed [8]. However, several studies have demonstrated a significant negative correlation between serum 25(OH)D levels and the activity of RA [6, 9–12]. Moreover, no correlation between serum 25(OH)D levels and the activity of RA was also observed in some reports [13–15]. Although these controversies still have not shown whether 25(OH)D deficiency is a primary phenomenon or an outcome of RA, these findings will help to understand the association between 25(OH)D and disease activity of RA. Thus, the objective of this study was performed to analyze the 25(OH)D levels in RA patients and healthy people and investigate the association between the serum levels of 25(OH)D_3_ and disease activity of RA patients from Southwest China.

## 2. Methods

### 2.1. General Data

RA patients (*n* = 116) and normal people (*n* = 50) were collected and admitted to Department of Rheumatology and Immunology, West China Hospital of Sichuan University, from June 2013 to December 2013. Participants were initially screened by telephone, and full assessments were conducted only for participants who did not report any exclusion criteria during the telephone screening. All participants provided written informed consent approved by the Hospital Human Research Ethics Committee of West China Hospital. All the cases included in the research were checked in by strict diagnosis standard. They were divided into two groups, the normal (*n* = 50) and RA (*n* = 116) group. The RA group includes 93 female and 23 male patients with RA diagnosed by Department of Rheumatology and Immunology, West China Hospital of Sichuan University. The control group includes 40 females and 10 males, which has no significant differences on sex and age with the RA group.

### 2.2. Inclusion and Exclusion Standards

Patients with RA in the clinical trials should be up to the standard as follows: (1) those who meet the diagnosis standard and modified classification of the American College of Rheumatology (ACR, 1987) [16] and (2) those who are beyond 18 years old. Patients with any of the following phenotypes should be excluded out of the clinical trials: (1) those who do not meet the diagnostic and modified classification standard; (2) those who are not beyond 18 years old; (3) those who have systemic lupus erythematosus, Sjogren syndrome, and other connective diseases; (4) those who have a damage in cardiovascular system, lung, kidney, and/or other organs.

### 2.3. Baseline Analysis

Clinical data of patients in the RA group were collected, including gender, age, height, weight, course of disease, swollen joint count, tender joint count, duration of morning stiffness, patient activity scale, C reactive protein (CRP), erythrocyte sedimentation rate (ESR), and rheumatoid factor (RF). Body mass index (BMI) and Disease Activity Score-28 (DAS28) of RA patients were calculated. Basic data of participants in the control group were collected, including gender, age, weight, and height, and BMI of each participant was calculated.

### 2.4. Analysis of Serum 25(OH)D Concentration

3 mL fasting blood samples of each participant in the RA and normal groups were centrifuged. Serum was applied for the measurement of 25(OH)D concentration using enzyme-linked immunosorbent (ELISA) method. The vitamin D kit of the British Immunodiagnostic Systems (IDS) was used. Correlation of 25(OH)D and disease activity of RA in the RA and control groups was analyzed. Moreover, RA group was divided into four subgroups according to DAS28 score, and further explore the association of between the values of DAS28 score and 25(OH)D levels in RA patients. According to the 25(OH)D levels, patients of RA were divided into reduced group (<50 nmol/L) and normal group (>50 nmol/L). The association between clinical data and disease activity in two groups was analyzed to explore the correlation of these parameters and 25(OH)D level.

### 2.5. Statistical Analysis

Statistical analysis was carried out using SPSS Version 19.0 for Windows. Data are presented as mean or median. The continuous variables that fit the Gaussian distribution were compared by using the independent samples *t* test, and the other parameters are compared by using the chi-square test. *P* values lower than 0.05 were regarded as statistically significant difference.

## 3. Results

### 3.1. Baseline Characteristics

Participant characteristics of patients in the RA and control groups were shown in Table 1. In the RA group, a total of 116 patients with the average age of 50.1 ± 10.9 years were included in this study, and the ratio of male and female was 1 : 4.04. The average height was 160.9 ± 6.4 cm, the average body weight was 57.5 ± 9 kg, and the mean body mass index (BMI) was 22.2 ± 2.9 kg/m^2^. In the control group, their average age was 48.1 ± 10.3 years, the ratio of male and female was 1 : 4, their average height was 161.6 ± 6.1 cm, the average body weight was 58.1 ± 8 kg, and the mean BMI was 22.2 ± 2.1 kg/m^2^. These results showed that there were no reliable differences in age, gender, height, body weight, and BMI in two groups (*P* > 0.05).

### 3.2. 25(OH)D Levels in the RA and Control Groups

Table 2 presents the clinical characteristics of the study subjects according to the level of 25(OH)D. As shown in Table 2, 25(OH)D levels of the peripheral blood in the RA groups were lower than those in the control groups (*P* < 0.05). The percentage of 25(OH)D less than 25 nmol/L in the RA groups was significantly higher than those in the control groups (22.4% versus 0,  *P* < 0.05, Table 2). The percentage of 25(OH)D from 25 to less than 50 nmol/L was 2.13-fold higher in the RA groups than those in the control groups (63.8% versus 20%,  *P* < 0.05, Table 2). However, the percentage of 25(OH)D with higher than 50 nmol/L in the RA groups was significantly lower than those in the control groups (13.8% versus 70%,  *P* < 0.05, Table 2). These results indicated that the percentage of patients with vitamin D inadequate and deficiency in the RA group was significantly higher than those in the control group, and there were statistically significant differences (*P* < 0.05).

### 3.3. 25(OH)D Levels in the RA Subgroups

Based on the Disease Activity Score-28 (DAS28), these patients with RA were stratified into four subgroups as follows: complete remission (DSA28 < 2.6), low disease activity (2.6 ≤ DAS28 < 3.2), moderate disease activity (3.2 ≤ DAS28 ≤ 5.1), and high disease activity (DAS28 > 5.1).  Table 3 shows the differences of 25(OH)D levels in peripheral blood of four subgroups patients. As shown in Table 3, the 25(OH)D levels of complete remission, low disease activity, middle disease activity, and high disease activity groups were 32.86 ± 12.26, 33.97 ± 13.28, 38.41 ± 10.64, and 38.94 ± 13.35 nmol/L, respectively. No significant differences were observed between complete remission and low disease activity group. Similar results were also found between middle disease activity and high disease activity group.

### 3.4. Comparison Analysis of RA Patients with 25(OH)D Deficiency and Normal Level Groups

Based on the 25(OH)D levels, these patients with RA were divided into 25(OH)D deficiency (*n* = 100) and normal (*n* = 16) groups.  Table 4 showed the results of comparison analysis of RA patients with 25(OH)D deficiency and normal groups. As shown in Table 4, there were no significant differences in age, sex, height, weight, BMI, disease duration, swelling and tenderness joint count, duration of morning stiffness, visual analogue scale (VAS), C-reactive protein (CRP), erythrocyte sedimentation rate (ESR), rheumatoid factor (RF), and DAS28 score (*P* > 0.05).

## 4. Discussion

RA is a chronic inflammatory autoimmune disease, mainly involving joint synovial membrane causing joint deformities. RA may cause bone loss and damage around joint [4, 5]. Reports have indicated that 90% of patients with RA symptoms will develop the radiological changes of joints damage during two years [17]. Studies have also found that T cells and cytokines play an important role in the pathogenesis of RA [18]. 25(OH)D is one of essential steroid hormones, which is involved in bone metabolism and immune regulation. Vitamin D receptors exist on the T and B lymphocytes, macrophages, and dendritic cells. After 25(OH)D combined with these receptors, they will regulate cell proliferation and differentiation and further inhibit the release of inflammatory factors. Then, they will play important roles in regulation of immune responses [19]. Thus, 25(OH)D may not only improve osteoporosis symptom of patients with rheumatoid arthritis but also play an important role in the regulation of immune system.

There has been a rapid growth in interest in the role of vitamin D in health and disease. 25(OH)D levels have been studied in RA. Previous reports have shown that serum 25(OH)D deficiency and inadequate is found in about 34–84% and 12–52% of RA patients, respectively [6, 10–12, 14, 15]. Several studies have also shown that serum 25(OH)D levels of patients with RA are significantly lower than those in the control group. Other studies reported that there were no significant differences in serum levels of 25(OH)D_3_ between RA patients and healthy controls [12, 20, 21]. These findings suggested that the proportion of patients with 25(OH)D deficiency and inadequate may be variable and related to population race, region, diet, sample size, age, sex, BMI, and other factors. In the present study, our results demonstrated that the percentages of patients with 25(OH)D deficiency and inadequate in RA group are 63.8% and 22.4%, respectively. This is close to the results of Furuya et al. [11]. Moreover, our results also showed that the serum 25(OH)D levels were significantly lower in tested RA patients than those in healthy controls. There were no significant differences in age, sex, height, weight, BMI, and so forth, showing that the lower level of serum 25(OH)D_3_ may be one of risk factors in the pathogenesis of rheumatoid arthritis.

In the previous study, the association between 25(OH)D levels and disease activity of rheumatoid arthritis still remains controversial. This may stem from the fact that 25(OH)D values are easily affected by population race, region, diet, sample size, age, sex, BMI, and other factors. Some studies confirmed that the reduction of serum 25(OH)D in patients of RA was negatively correlated with clinical indexes (joint swelling, joint tenderness, ESR, etc.) and disease activity index DAS28 [6, 9–12]. However, some reports indicated that there was no relation between 25(OH)D deficiency and disease activity in RA [13–15]. The present study suggested that no statistical difference was found among four subgroups in the RA group based on the classification of DAS28 value. Moreover, our findings also indicated that no significance between 25(OH)D deficiency and normal level groups was found under the comparison analysis of clinical parameters and DAS28 scores in these RA patients. Based on the above results, the present findings indicated that serum 25(OH)D levels might be not associated with the disease activity of RA.

## 5. Conclusion

In summary, the present study suggested that serum 25(OH)D level might be significantly associated with the pathogenesis of RA. However, the serum values of 25(OH)D are not a good indicator of disease activity of RA patients. As serum 25(OH)D_3_ levels are not independent and/or influenced by many factors, such as region, season, and gender, further research will focus on the cross-regional and large sample to understand the association between 25(OH)D and RA in Southwest China.

## Figures and Tables

**Table 1:** Participant characteristics of patients in the RA and control groups.

Variable	RA	Controls	*P* value
Age (years)	50.1 ± 10.9	48.1 ± 10.3	0.283
Ratio of female : male	93 : 23	40 : 10	0.980
Weight (kg)	57.5 ± 9.0	58.1 ± 8.0	0.686
Height (cm)	160.9 ± 6.4	161.6 ± 6.1	0.568
BMI (kg/m^2^)	22.2 ± 2.9	22.2 ± 2.1	0.970

**Table 2:** Distribution of 25-hydroxyvitamin D level in the RA and control groups.

25(OH)D level	RA (*n* = 116)	Controls (*n* = 50)	*P* value
Tested level (nmol/L)	35.99 ± 12.59	54.35 ± 8.20	<0.05
Deficiency (≤25 nmol/L)	26 (22.4%)	0 (0)	<0.05
Insufficiency (25–50 nmol/L)	74 (63.8%)	15 (30%)	<0.05
Normal level (≥50 nmol/L)	16 (13.8%)	35 (70%)	<0.05

**Table 3:** Differences of level of 25(OH)D in peripheral blood of four subgroups in the RA group.

Subgroups	Vitamin D level (nmol/L)	*P* value^∗^
DAS28 < 2.6	32.86 ± 12.26	>0.05
2.6 ≤ DAS28 < 3.2	33.97 ± 13.28	>0.05
3.2 ≤ DAS28 ≤ 5.1	38.94 ± 13.35	>0.05
DAS28 > 5.1	38.41 ± 10.64	>0.05

^∗^ *P* values were obtained from the comparison of 25(OH)D levels among the subgroups.

**Table 4:** Comparison analysis of RA patients with 25(OH)D deficiency and normal levels groups (<50 nmol/L).

Characteristics	Deficiency (*n* = 100)	Normal level (*n* = 16)	*P* value
Age (years)	49.8 ± 10.8	52.3 ± 11.7	0.684
Female : male ratio	81 : 19	12 : 4	0.580
Weight (kg)	57.5 ± 8.7	58.0 ± 10.9	0.608
Height (cm)	160.7 ± 6.2	162.2 ± 7.8	0.503
BMI (kg/m^2^)	22.2 ± 3.0	21.9 ± 2.3	0.149
Disease duration (months)	43.5 (3, 468)	36.0 (3, 316)	0.957
DAS28	3.5 ± 1.8	4.09 ± 1.6	0.779
Patient global VAS (cm)	3.3 ± 3.1	4.2 ± 3.1	0.948
Swollen joint count (0–28)	3.9 ± 5.4	5.3 ± 7.1	0.302
Tender joint count (0–28)	5.1 ± 6.5	6.9 ± 7.1	0.775
ESR (mm/H)	33.5 (4, 102)	36.5 (6, 78)	0.360
CRP (mg/L)	5.1 (1, 78)	5.7 (3.2, 17.7)	0.595
RF (IU/ml)	63.5 (20, 2220)	107.5 (20, 1900)	0.009
